# Improving the antiprotozoal effect of saponins in the rumen by combination with glycosidase inhibiting iminosugars or by modification of their chemical structure

**DOI:** 10.1371/journal.pone.0184517

**Published:** 2017-09-08

**Authors:** Eva Ramos-Morales, Gabriel de la Fuente, Robert J. Nash, Radek Braganca, Stephane Duval, Marc E. Bouillon, Martina Lahmann, C. Jamie Newbold

**Affiliations:** 1 Institute of Biological, Environmental and Rural Sciences, Aberystwyth University, Aberystwyth, United Kingdom; 2 PhytoQuest Ltd, Aberystwyth, United Kingdom; 3 BioComposites Centre, Bangor University, Bangor, United Kingdom; 4 DSM Nutritional Products Ltd., Centre de Recherche en Nutrition Animale, Saint Louis Cedex, France; 5 School of Chemistry, Bangor University, Bangor, United Kingdom; University of British Columbia, CANADA

## Abstract

The antiprotozoal effect of saponins is transitory, as when saponins are deglycosylated to sapogenins by rumen microorganisms they become inactive. We hypothesised that the combination of saponins with glycosidase-inhibiting iminosugars might potentially increase the effectiveness of saponins over time by preventing their deglycosylation in the rumen. Alternatively, modifying the structure of the saponins by substituting the sugar moiety with other small polar residues might maintain their activity as the sugar substitute would not be enzymatically cleaved. The aim of this *in vitro* study was to evaluate the acute antiprotozoal effect and the stability of this effect over a 24 h incubation period using ivy saponins, a stevia extract rich in iminosugars, ivy saponins with stevia extract, and a chemically modified ivy saponin, hederagenin *bis*-succinate (HBS). The effects on fermentation parameters and rumen bacterial communities were also studied. Ivy saponins with stevia and HBS had a greater antiprotozoal effect than ivy saponins, and this effect was maintained after 24 h of incubation (P<0.001). The combination of ivy and stevia extracts was more effective in shifting the fermentation pattern towards higher propionate (+39%) and lower butyrate (-32%) and lower ammonia concentration (-64%) than the extracts incubated separately. HBS caused a decrease in butyrate (-45%) and an increase in propionate (+43%) molar proportions. However, the decrease in ammonia concentration (-42%) observed in the presence of HBS was less than that caused by ivy saponins, either alone or with stevia. Whereas HBS and stevia impacted on bacterial population in terms of community structure, only HBS had an effect in terms of biodiversity (P<0.05). It was concluded that ivy saponins with stevia and the modified saponin HBS had a strong antiprotozoal effect, although they differed in their effects on fermentation parameters and bacteria communities. Ivy saponins combined with an iminosugar-rich stevia extract and/or HBS should be evaluated to determine their antiprotozoal effect *in vivo*.

## Introduction

Manipulation of the rumen microbial ecosystem to increase the efficiency of nutrient utilization and reduce the environmental impact of ruminant husbandry has long been a target for rumen nutritionists and microbiologists. The elimination of the ciliate protozoa has been shown to increase microbial protein supply to the host by up to 30% and reduce methane production by up to 11% [1]. In recent years there has been an increased interest in the use of plant secondary metabolites as antiprotozoal agents with a particular emphasis on saponins [2].

Saponins consist of a sugar moiety (e.g. D-glucose, D-galactose, D-glucuronic acid, D-xylose, L-rhamnose) glycosidically linked to a hydrophobic aglycone, the sapogenin [3]. The antiprotozoal effect of saponins is related to their interaction with the sterol moiety present in the membrane of protozoa [4]. Rumen protozoa species differ in their sensitivity to saponins due to different composition of sterols in their membrane [4]. The antiprotozoal effect of saponins seems to be transitory as when saponins are deglycosylated to their sapogenins by rumen microorganisms they become inactive [4,5] which represents a challenge to their practical application in ruminant nutrition.

*Stevia rebaudiana* Bertoni (Asteraceae) has been used as a natural sweetener due to its high content of the sweet-tasting glycosides of the diterpene derivative steviol [6]. Stevia also contains iminosugars [7], a class of compounds well known for their ability to inhibit glycosidases involved in a wide range of important biological processes [8,9]. Thus, an iminosugar rich stevia extract might increase the effectiveness of saponins by preventing their deglycosylation in the rumen. The major iminosugar in stevia leaves is the glycosidase inhibitor 2,5-dihydroxymethyl-3,4-dihydroxypyrrolidine (DMDP) that is present at 0.1 to 1% dry matter (DM). Preliminary *in vitro* studies [10], combining a saponin extract with DMDP have shown the potential of this strategy to maintain saponin activity over 24 h. We also hypothesised that modifying the structure of the saponin by substituting the sugar moiety with other small polar residues might preserve its activity as the sugar substitute would not be enzymatically cleaved by glycosidases [11].

The aim of this *in vitro* study was to evaluate the effects on fermentation parameters and rumen bacteria communities, the acute antiprotozoal effect and the stability of this effect over 24 h, comparing ivy saponins, with or without a stevia extract rich in iminosugars, with a chemically modified ivy saponin (HBS).

## Material and methods

### Ivy and stevia extracts and hederagenin *bis*-succinate

Ripe ivy (*Hedera helix*, Araliaceae) fruits were botanically identified and collected by D. Preskett (Bangor University) from several locations around Bangor (44.8036° N, 68.7703° W, UK), dried at 50°C for two days and milled. Ivy fruit meal (3.79 kg) was extracted with ethanol (15 L) for 6 h, leading to a crude extract (541 g) comprising triglycerides, saponins, oligosaccharides, and pigments (anthocyanins). The crude extract was then washed with petroleum ether (3 × 500 mL) and dried overnight at 50°C under vacuum, obtaining a fine powder (368 g) which contained mainly hederagenin constituted saponins and oligosaccharides. Then an additional extraction with *n*-butanol was carried out, producing a refined extract comprising saponins (15% DM). Hederagenin, the aglycone part of the saponins, was obtained via hydrolysis of ivy fruit refined extract in ethanolic solution with aqueous HCl. HBS was synthetized from the aglycone hederagenin according to synthetic methods for esterification of organic molecules [12] as described in patent application PCT/EP2016062383 (“New bis esters of Ivy sapogenins for ruminants”) [13]. Stevia extract was obtained from dried and milled leaves of *Stevia rebaudiana* Bertoni after extraction with 50% ethanol for 15 h at 200 mg/mL and then filtering and vacuum drying. Analysis of the iminosugars in the stevia extract was conducted by gas chromatography-mass spectroscopy (GCMS) of the trimethylsilyl-derivatives after cation exchange chromatography [14]. The extract obtained was particularly rich in DMDP (0.2%) and other iminosugars (0.1%). Ivy fruit and stevia leaves extracts were provided by Bangor University and PhytoQuest Ltd, respectively. HBS was synthetized at Bangor University and DSM Nutritional Products Ltd.

### Measurement of protozoal activity

The effect of ivy and stevia extracts and HBS on protozoal activity was measured *in vitro* as the breakdown of [^14^C] labelled bacteria by rumen protozoa as described by Wallace and McPherson [15]. Isotope-labelled bacteria were obtained by growing *Streptococcus bovis* in Wallace and McPherson media [15] containing [^14^C] leucine (1.89 μ Ci/7.5 mL tube) for 24 h. Cultures were centrifuged (3,000 × *g* for 15 min), supernatant discarded and pellets re-suspended in simplex type salt solution (STS) [16] containing ^12^C-leucine (5 mM). This washing process was repeated three times. The labelled bacterial suspension was sampled to determine its radioactivity and then it was used as inoculum in the incubations with rumen fluid.

Rumen digesta was obtained from four rumen-cannulated Holstein-Frisian cows (4 replicates), fed at maintenance level (diet composed of perennial ryegrass hay and concentrate in a 67:33 ratio on DM basis). Animal procedures were carried out according to the UK Home Office Scientific Procedures Act 1986 (PLL 40/3653; PIL I90661131) and protocols were approved (July 02, 2015) by the Aberystwyth University Ethical Committee. Rumen digesta was obtained before the morning feeding and strained through two layers of muslin and diluted with STS (1:1). Diluted rumen fluid (7.5 mL) was then incubated with labelled bacteria (0.5 mL) in tubes containing no additive (control) or 0.5, 1 or 2 g/L of ivy extract, stevia extract or HBS. HBS was solubilized in ethanol at 1% of the incubation volume as it has been shown that such concentration of ethanol in rumen fluid should not impair fermentation [17,18]. A control treatment with 1% of ethanol was also included in the experimental design.

Incubations were carried out at 39°C under CO_2_, and tubes were sampled at time 0 and at 1 h intervals up to 5 h using a syringe with a 19-gauge needle. Samples (0.5 mL) were acidified (by adding 125 μL of trichloroacetic acid at 25% wt/vol) and centrifuged (13,000 × *g* for 5 min). Supernatant (200 μL), was diluted with 2 mL of scintillation fluid to determine the radioactivity released by liquid-scintillation spectrometry (Hidex 300 SL, Lablogic Systems Ltd, Broomhill, UK). Bacterial breakdown at each incubation time was expressed as the percentage of the acid-soluble radioactivity released relative to the total radioactivity present in the initial labelled bacteria [15].

### In vitro batch cultures

To evaluate the stability of the antiprotozoal effect over 24 h and to study the effect of ivy saponins, with or without stevia iminosugars, and HBS on fermentation parameters, strained rumen fluid from each cow was diluted 1:2 in artificial saliva [19]. Aliquots (30 mL) of the diluted strained rumen fluid were added anaerobically to 120 mL serum bottles (Sigma-Aldrich Ltd, Dorset, UK) containing 0.3 g of diet composed of ryegrass hay and barley (40:60 ratio on a DM basis), previously ground to pass through a 1-mm^2^ mesh screen. Treatments consisted of control incubations (0.3 g of only diet) and incubations with either ivy (1 g/L) or stevia (2 g/L) extracts alone or combined (1 g/L of ivy refined extract + 2 g/L of stevia extract) or HBS (1 g/L). Bottles were incubated at 39°C under CO_2_ receiving a gentle mix before each sampling time. Samples at different time points (0, 4, 8 and 24 h) were collected for visual assessment of protozoa motility. Ciliate protozoa motility was assessed in 30 μL of sample against a common scale when examined at low magnification (× 100) using light microscopy. This evaluation was conducted in less than 1 min/sample to avoid the cell damage caused by exposure to oxygen and low temperature. A score between 0 (no whole protozoa evident) and 5 (all genera active) was given according to the scale described by Newbold [20].

Fermentation pattern, in terms of pH, ammonia and volatile fatty acids (VFA) was determined after 24 h of the incubation. A subsample (4 mL) of incubation fluid was diluted with 1 mL of deproteinising solution (200 mL/L orthophosphoric acid containing 20 mmol/L of 2-ethylbutyric acid as an internal standard) for the determination of VFA using GC, as described by Stewart and Duncan [21]. Another subsample (1 mL) was diluted with 0.250 mL of trichloroacetate (25% (wt/vol)) for analysis of ammonia using a colorimetric method [22]. Additionally, at the end of the incubation period, samples for protozoa counting and DNA extraction prior to bacteria community structure analysis were collected. For protozoa counting, samples (0.5 mL) were collected in 0.5 mL of saline formalin solution (4% formaldehyde and 0.9% NaCl in distilled water). For DNA extraction, samples (2 mL) were preserved in RNA-later stabilization solution (8 mL; Qiagen Ltd, West Sussex, UK) and stored overnight at 4°C.

Protozoa were quantified by optical microscope using the procedure described by Dehority [23] and adapted by de la Fuente et al. [24]. Ciliates were quantitatively classified in terms of *Isotricha* sp. and *Dasytricha* sp. representatives of the holotrich protozoa, and by the subfamily Entodiniinae, subfamily Diplodiniinae and subfamily Ophryoscolecinae, as representatives of entodiniomorphid protozoa. DNA extraction was conducted according to Pitchern et al. [25] and Boom et al. [26], with modifications. Guanidium hydrochloride instead of guanidium thiocyanate was used combined with EDTA and sarkosyl, together with silica particles.

Rumen bacteria communities were studied using T-RFLP analysis. PCR was performed using a 16S rRNA bacterial-specific primer pair, cyanine-labelled 27F (5′-AGA GTT TGA TCC TGG CTG AG-3′) and unlabelled 1389R (5′-AGG GGG GGT GTG TAG AAG-3′) [27] following Skrivanová et al. [28]. A 25 μL reaction was prepared containing 1.25 U GoTaq DNA polymerase (Promega UK Ltd., Southampton, UK), 1 × Promega reaction buffer, 1.75 mM MgCl_2_, 0.2 mM of each dNTP with each primer used at 0.5 μM. Resultant amplicons were analysed on a 1% (w/v) TAE agarose gel to assess the quality of amplification.

DNA concentration of each amplified and purified sample was determined by spectrophotometry (Nanodrop ND-1000 spectrophotometer) to enable a standardised quantity of 50 ng DNA to be used for digestion with restriction enzymes. Digestion of samples was carried out using the restriction enzymes, HhaI, HaeIII, and MspI (New England Biolabs UK Ltd.) following the manufacturers recommendations with the exception of HhaI where the recommended addition of bovine serum albumin was omitted.

Restriction digests (20 μL) were purified by ethanol precipitation in a thermowell 96-well PCR plate (Costar; Corning Inc., NY). DNA was precipitated by adding 120 μL of 95% ethanol at −80°C, 4 μL of EDTA (100 mM), 4 μL of sodium acetate (3M, pH 5.2) and 4 μL of glycogen (20 mg/mL), followed by centrifugation for 30 min at 4°C at 3,000 *g*. DNA pellets were washed twice with 200 μL of 70% ethanol, air-dried at room temperature and re-suspended in 35 μL sample loading solution buffer including a 600 bp size standard (Beckman Coulter Inc., Fullerton). T-RFs were separated on a CEQ 8000 Genetic Analysis System (Beckman Coulter, High Wycombe, UK) using the Frag4 parameters (denaturation step at 90°C for 120 seconds; injection at 2 kV for 30 seconds; separation at 4.8 kV for 60 min with a capillary temperature of 50°C). The protocol and software used was as described by Skrivanová et al. [28] using the Local Southern method to distinguish true peaks from background noise. The following criteria were applied prior to exporting data from the CEQ 8000 genetic analysis system: Slope threshold of 5 and relative peak height of 5% (where 5% of the second highest peak was used as the lower threshold for peak identification). These parameters allow detection and elimination of smaller, broader peaks that would have a less specific size and would not be indicative of single true OTUs.

### Calculations and statistical analysis

A simple linear regression was conducted to model the relationship between the percentage of radioactivity released (relative to the ^14^C-bacterial inoculum) and the time (from 0 h to 5 h), as well as its correlation coefficient. The slope of this trend-line indicated the bacterial degradation rate (as % h^-1^) and ultimately acts as a proxy of protozoal activity. Trend line slopes and fermentation parameters were analysed statistically by randomized block ANOVA, with individual cows as a blocking term. When significant effects were detected across the different doses, means were compared by Fisher’s unprotected LSD test. For the rates of bacterial degradation, polynomial contrasts were also used to determine linear (L) and/or quadratic (Q) responses to the treatments. Richness, Shannon-Wiener and Simpson diversity indexes were calculated using normalised fragment data [29] and analysed by one-way ANOVA as above.

Protozoal motility was analysed as a repeated measurements design, with treatment as the main factor and incubation time as the subject factor. Interaction between treatment and time as a measure of differential temporal dynamics between treatments was also considered. Differences were declared significant at P<0.05 and considered as tendencies towards significance at P<0.10. All analyses were carried out using Genstat 15th Edition (VSN International, Hemel Hempstead, UK).

The overall treatment effect on the T-RFLP data was also tested using Permutational Analysis of Variance (Permanova). Pairwise comparisons were conducted to elucidate differences between treatments. The pseudo-F statistics and P-values were calculated after 5,000 random permutations of residuals under a reduced model using the Monte Carlo test. P values were adjusted for multiple testing using the method proposed by Benjamini and Hochberg [30] to decrease the False Discovery Rate.

Main effects of pH, ammonia, VFA and protozoa concentration were analysed in the community structure using Canonical Correspondence Analysis (CCA) using R statistical program and the package “vegan” under the formula: Y = Ac + Pr + Bu + Bc + pH + Am + Prot, where Y was the T-RFLP matrix data, Ac, Pr, Bu, Bc and Am were the molar concentrations of acetic acid, propionic acid, butyric acid, BCVFA and ammonia, respectively, and Prot was the logarithmic concentration of total protozoa in the incubations. Permanova was also used to elucidate the specific effect of the metabolites included in the CCA model.

## Results

### Antiprotozoal activity

Bacterial degradation by protozoa increased linearly (R^2^>0.99) over the 5 h incubation with both control and control plus ethanol treatments. The rate of bacterial breakdown was significantly lower (P<0.001) in the presence of ivy and stevia extracts and HBS, at all the concentrations tested, as compared to the control (Table 1). Increasing levels of ivy extract resulted in a linear and quadratic decrease (P<0.001) in the breakdown of bacteria by protozoa. Stevia extract promoted a linear decrease (P<0.001) in protozoa activity. HBS caused a dramatic reduction in protozoa activity (P<0.001) with no bacterial breakdown observed when incubated at 1 and 2 g/L.

**Table 1:** Effect of ivy and stevia extracts and HBS, added at 0.5, 1 or 2 g/L, on rumen protozoa activity assessed *in vitro* as the amount of ^14^C-labelled bacteria broken down by rumen protozoa (% of the initial radioactivity released per hour).

	Dose (g/L)						
Treatment	0	0.5	1	2	SED	P	Contrast
Ivy	4.12^c^	1.68^b^	0.60^a^	0.27^a^	0.414	<0.001	L*,Q*
Stevia	4.12^d^	3.28^c^	2.35^b^	0.67^a^	0.328	<0.001	L*
HBS	5.30^b^	0.48^a^	0^a^	0^a^	0.302	<0.001	-

^a-d^ Means with different superscript differ (*n* = 4); L: linear response; Q: quadratic response;

*:P<0.001

### Stability of the antiprotozoal effect and effect on fermentation parameters

Based on the observed effects of ivy and stevia extracts and HBS on bacterial breakdown by protozoa, the effect of selected doses of the plant extracts and the synthesised compound were tested in 24 h incubations. Ivy extract and HBS were incubated at 1 g/L and stevia extract at 2 g/L, either on its own or combined with 1 g/L of ivy extract. Protozoa motility over time was assessed and fermentation parameters and protozoa numbers were determined after 24 h of incubation.

When total protozoal numbers after 24 h were assessed (Table 2), ivy extract reduced protozoa numbers by 56% and stevia, ivy+stevia and HBS treatments caused a reduction of 76–80% (P<0.001). Differences in the relative abundance of the main protozoal groups between treatments were not detected (P>0.05), probably due to the high level of variation between replicates. Nevertheless, ivy+stevia and HBS treatments seemed to promote a greater percentage of *Entodinium* and lower abundance of *Diplodinium*, compared with the rest of the treatments. No holotrichs were present in incubations with ivy+stevia or HBS. Stevia extract, either alone or combined with ivy, appeared to increase the relative proportion of *Epidinium*.

**Table 2:** Effect of ivy (1 g/L) and stevia (2 g/L) extracts, either alone or combined, and HBS (1 g/L) on protozoa in ruminal digesta after 24 h of incubation.

	Treatments						
	Control	Ivy	Stevia	Ivy+Stevia	HBS	SED	P
Total (log cells/mL)	4.97^c^	4.61^b^	4.26^a^	4.30^a^	4.35^a^	0.09	<0.001
*Entodinium* %	52.9	49.2	48.1	55.7	64.8	7.54	0.246
*Epidinium* %	2.8	3.4	8.8	8.9	3.2	2.81	0.095
*Diplodinium* %	42.5	46.1	40.7	35.4	32	7.92	0.444
*Isotricha* %	0.7	0.8	1.3	0	0	1.03	0.663
*Dasytricha* %	1.1	0.5	1.0	0	0	0.85	0.543

^a-c^ Means with different superscript differ (*n* = 4).

**Fig 1:**
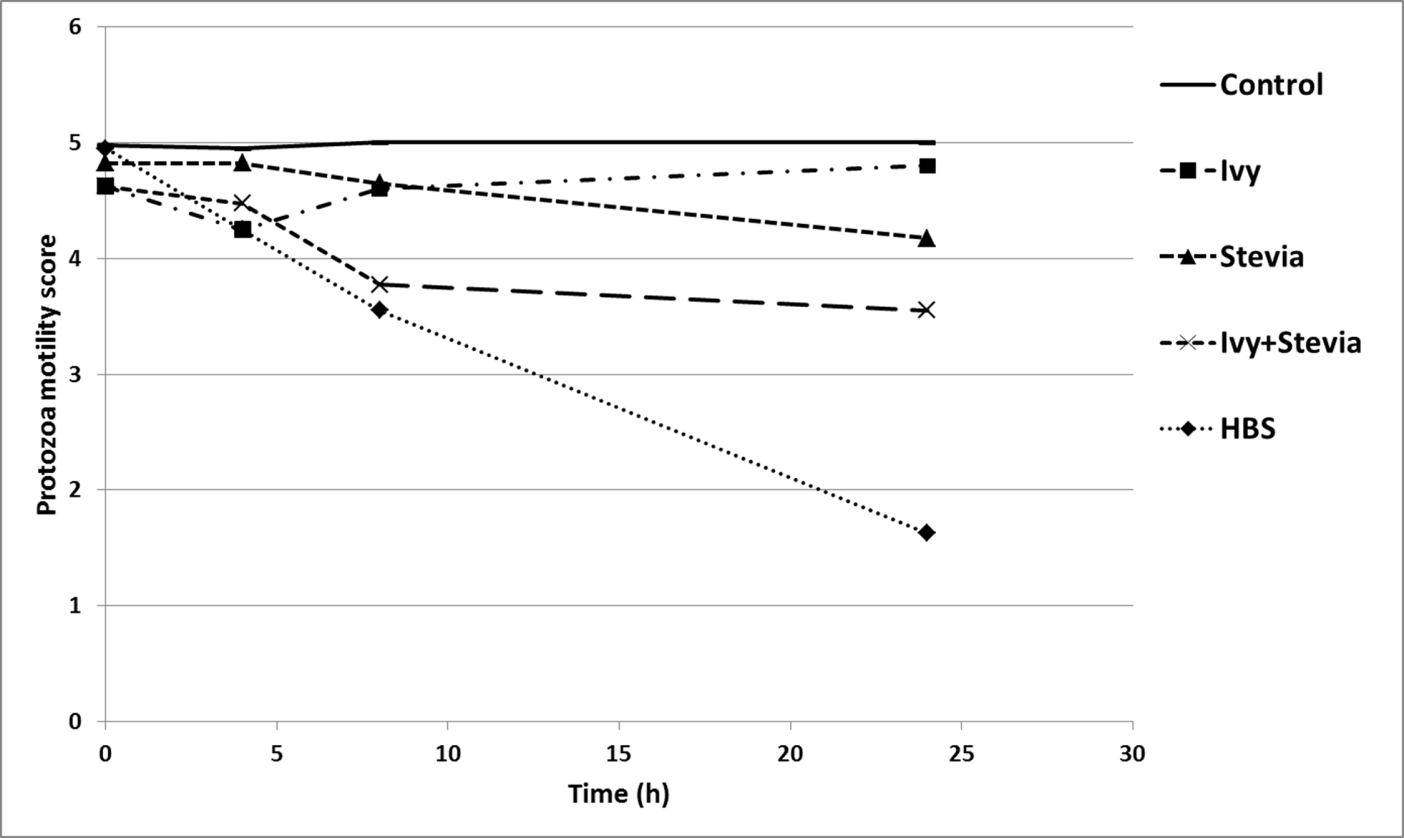
Effect of ivy (1 g/L) and stevia (2 g/L) extracts, either alone or combined, and HBS (1 g/L) on protozoa motility over 24 h *in vitro* incubations.

Cell motility, measured as an index of protozoa viability, remained unaltered (score of 5) over the 24 h incubation period in control incubations (Fig 1). Although the addition of ivy extract decreased protozoa motility at 4 h of the incubation (score of 4.25; P<0.001), motility recovered afterwards (reaching a score of 4.8 at 24 h; treatment × time interaction, P<0.001) suggesting, as expected, degradation of the saponin during incubation. However, the combination of ivy with stevia caused a slight decrease in protozoa motility at 4 h that was more pronounced at 8 h (scores of 4.78 and 3.78, respectively), with no recovery after 24 h (score of 3.55; treatment × time interaction, P<0.001). The greatest effect on protozoa motility was observed in the presence of HBS which caused a decrease in motility after 4 h with no sign of recovery. Indeed, vacuoles were visible at 24 h in most of the protozoa and some of them even showed cellular disruption (score of 1.6).

The addition of ivy and stevia extracts, either alone or combined, and HBS had a significant impact on rumen fermentation parameters (Table 3). All treatments caused a decrease (P<0.001) in pH and ammonia concentration compared to the control, with the combination of ivy and stevia having the greatest effect (ammonia concentration reduced by 64%). The concentration of VFA increased (P = 0.025) when ivy+stevia or HBS were incubated. All treatments caused a shift in the molar proportions of VFA towards higher propionate and lower butyrate and branched-chain VFA (BCVFA, i.e. isobutyrate and isovalerate) (P<0.001). The molar proportion of acetate was lower in incubations with ivy in comparison to the rest of the treatments (P = 0.002).

**Table 3:** Effect of ivy (1 g/L) and stevia (2 g/L) extracts, either alone or combined, and HBS (1 g/L) on pH, NH_3_-N and VFA profile in ruminal digesta after 24 h of incubation.

	Treatment						
	Control	Ivy	Stevia	Ivy+Stevia	HBS	SED	P
pH	6.33^d^	6.27^c^	6.17^b^	6.11^a^	6.17^b^	0.016	<0.001
NH_3_-N (mmol/L)	9.56^d^	4.16^b^	5.34^c^	3.39^a^	5.56^c^	0.24	<0.001
Total VFA (mmol/L)	98.0^a^	99.2^a^	105^a^^b^	111^b^	110^b^	4.18	0.025
VFA (mmol/mol) Acetate	654^b^^c^	627^a^	657^c^	643^b^	650^b^^c^	6.03	0.002
Propionate	171^a^	219^b^	217^b^	237^c^	246^d^	3.75	<0.001
Butyrate	134^d^	121^c^	94.0^b^	90.4^b^	75.8^a^	0.198	<0.001
BCVFA	21.0^d^	13.9^c^	13.9^c^	11.1^a^	12.5^b^	0.030	<0.001

^a-d^ Means with different superscript differ (*n* = 4).

### Effects on bacterial communities

**Fig 2:**
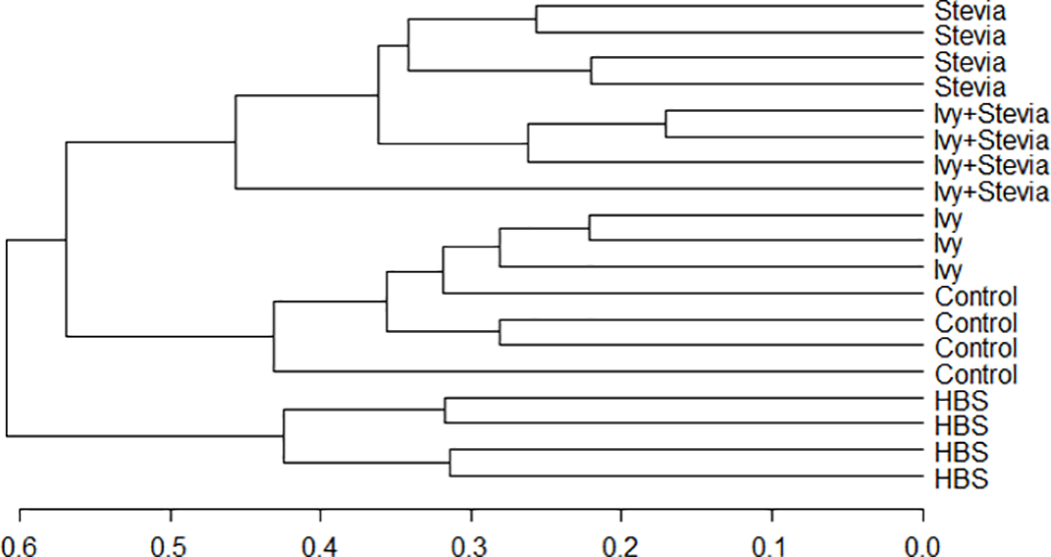
Dendrogram obtained from the TRFLP analysis of samples from incubations with no additive (Control), ivy, stevia, ivy+stevia and HBS. Scale bar show percentage of similarity.

T-RFLP generated 344 T-RFs from three restriction enzymes (HhaI, HaeIII, MspI). The dendrogram obtained from the T-RFLP analysis (Fig 2) showed two main clusters, separating HBS samples from those belonging to the rest of the treatments (60% similarity). Within the second cluster, samples belonging to stevia and ivy+stevia treatments clustered separately from those corresponding to ivy and control treatments. Permutational analysis of variance showed a significant effect of the treatments on the structure of the bacterial community (P = 0.001).

**Fig 3:**
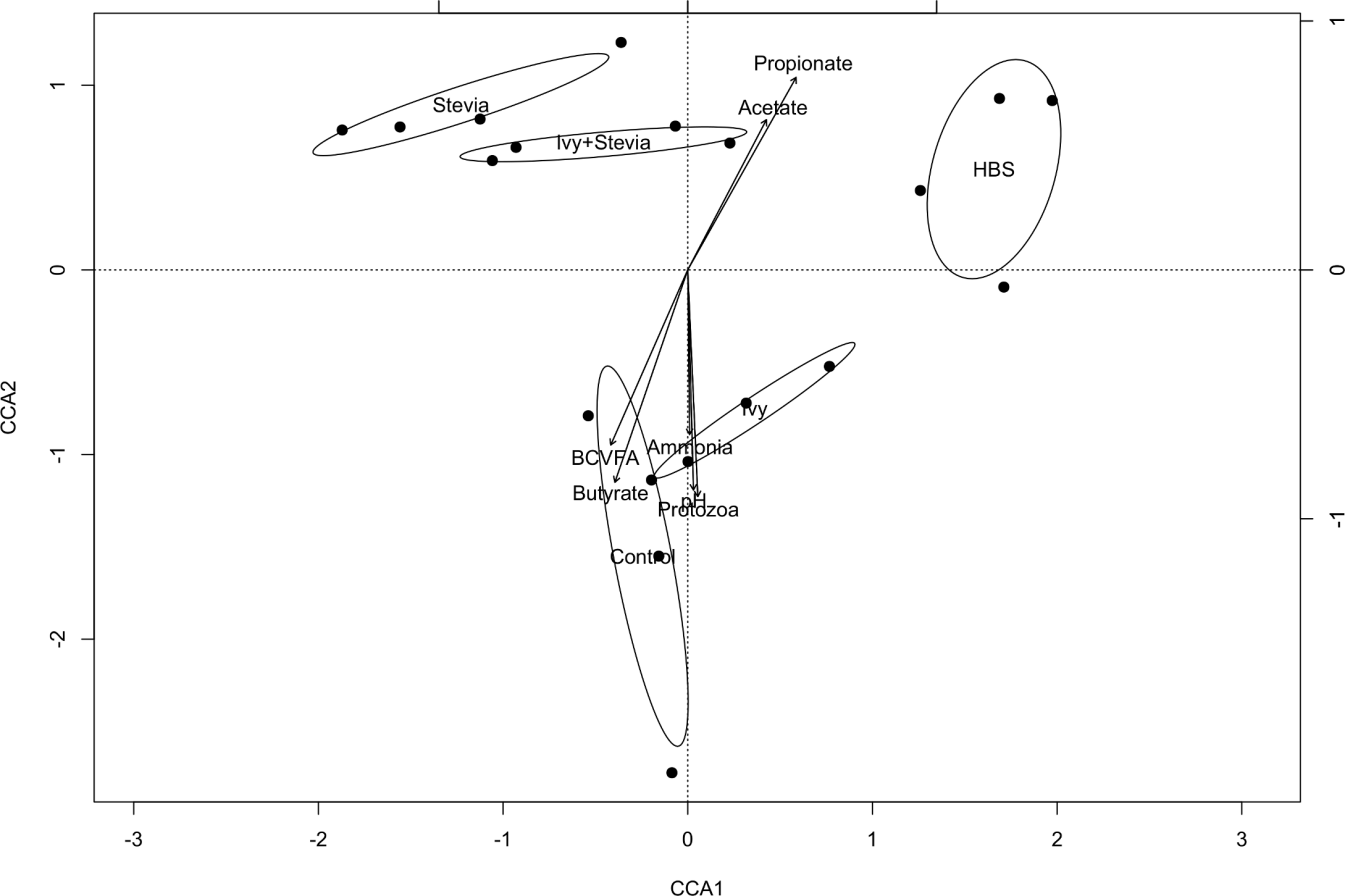
CCA illustrating the relationship between the structure of the bacterial community with the rumen fermentation pattern. Arrows show the direction of the gradient and their length is proportional to the correlation. Confidence interval (95%) is indicated for each treatment: control, ivy, stevia, ivy+stevia or HBS. CCA1: 26.08%; CCA2: 24.42%.

Pairwise analysis showed that the structure of the bacterial community differed (P<0.05) between treatments (Table 4), except in the case of control and ivy treatments (P = 0.135). In order to detect possible correlations between the structure of the bacterial community and rumen fermentation parameters, a Canonical Correspondence Analysis (CCA) was performed (Fig 3). There was a significant grouping effect (Permutation test for CCA, P = 0.035) with samples from HBS segregated from those of the control and ivy treatments and stevia and ivy+stevia treatments. Permutational tests also showed that protozoa (P = 0.44) and ammonia (P = 0.019) concentrations as well as butyrate (P = 0.136) and BCVFA (P = 0.39) molar proportions were negatively correlated to the structure of the bacterial community of HBS, stevia and ivy+stevia samples. Propionic (P = 0.044) and acetic acid (P = 0.007) were negatively correlated to the structure of the bacterial community of control and ivy samples (Fig 3).

**Table 4:** Effect of ivy (1 g/L) and stevia (2 g/L) extracts, either alone or combined, and HBS(1 g/L) on the structure of the bacterial communities.

Pairs	Similarity	F.Model	R^2^	P-adjusted
Control vs HBS	47.0	4.42	0.4240	0.033
Control vs stevia	43.9	4.24	0.4140	0.033
Control vs ivy	32.2	1.55	0.2370	0.135
Control vs ivy+stevia	41.9	3.31	0.3552	0.002
HBS vs stevia	51.5	7.45	0.5540	0.002
HBS vs ivy	38.8	3.60	0.4188	0.002
HBS vs ivy+stevia	46.9	5.16	0.4624	0.002
Stevia vs ivy	41.9	5.94	0.5429	0.031
Stevia vs ivy+stevia	37.5	2.22	0.2697	0.002
Ivy vs ivy+stevia	35.0	3.77	0.4299	0.002

Bacterial communities were studied using T-RFLP data. Permutational analysis of variance was performed using Bray–Curtis similarity measurements of number of fragments and peak height. Higher Pseudo-F and lower similarities and P-values correspond to greater differences in the microbial composition (n = 4).

Bacterial diversity indexes are shown in Table 5. Although no differences were detected in bacterial richness (P = 0.132), values were reduced by 15% and 39% with ivy+stevia and HBS treatments, as compared to the control. Both Shannon and Simpson indexes were significantly lower (P = 0.007 and P = 0.006, respectively) in incubations with HBS, as compared to the rest of the treatments.

**Table 5:** Effect of ivy (1 g/L) and stevia (2 g/L) extracts, either alone or combined, and HBS (1 g/L) on bacterial diversity in ruminal digesta after 24 h of incubation.

	Treatment						
	Control	Ivy	Stevia	Ivy+stevia	HBS	SED	P
Shannon index	4.30^b^	4.19^b^	4.39^b^	4.31^b^	3.52^a^	0.205	0.007
Simpson index	0.975^b^	0.971^b^	0.978^b^	0.979^b^	0.946^a^	0.008	0.006
Richness	144	135	140	122	88	21.87	0.132

^a-c^ Means with different superscript differ (*n* = 4).

## Discussion

Plants or their extracts with high concentrations of saponins appear to have the potential to act as natural rumen manipulators. The most commonly sources of saponins used in ruminant nutrition are *Yucca schidigera* (Asparagaceae), rich in sterol saponins, and *Quillaja saponaria* (Quillajaceae) which contains triterpene saponins, although other sources of saponins such as tea saponins (triterpenoid) have also been explored [31,32]. Their main effect is the suppression of rumen protozoa which has been consistently observed in *in vitro* studies [33]. This effect seems to be transitory because of the degradation of saponins by rumen bacteria [5] as when saponins are deglycosylated to sapogenins they are no longer active against rumen protozoa.

Common ivy or English ivy (*Hedera helix L*., Araliaceae) has been used as a medicinal plant due to its antimicrobial and antifungal properties [34,35]. The active constituents in ivy fruit include triterpene saponins that are mainly derived from hederagenin, as well as fatty acids and polyacetylenes [36]. Recent studies in our group have shown the potential of a crude extract from ivy fruit rich in saponins (15% in DM) to modulate rumen function [37,38]. In this study, we used a refined extract from ivy fruits, with the same saponin composition but with no oligosaccharides or triglycerides present. Our results showed that the ivy refined extract, had a strong antiprotozoal effect *in vitro* when measuring protozoal activity based on the amount of released [^14^C] from labelled bacteria. This observation was confirmed in 24 h batch cultures as protozoa numbers were reduced by 56% when 1 g/L of ivy refined extract was added. Protozoa motility over time was also affected although a recovery after 24 h was observed.

*In vitro* incubations with ivy extract resulted in decreased acetate (-4%) and butyrate (-10%) molar proportions and increased propionate (+28%), without affecting the concentration of total VFA, in comparison to the control. Additionally, BCVFA, which primarily originate from dietary protein or bacterial protein breakdown in the rumen [39], and ammonia concentration were reduced (by 34% and 56%, respectively) when ivy extract was incubated at 1 g/L. These changes in the fermentation pattern, typically of those observed when protozoal concentration is reduced, have been previously reported *in vitro* with other saponin sources [4,40]. Although reports are sometimes contradictory with the discrepancies being attributed to differences in the chemical structure and dosage of saponins used, diet composition, as well as adaptation of the microorganisms to saponins [4,33]. Generally, lower acetate to propionate ratios and protozoal counts have been accompanied by a reduction in methane production [40]. Stoichiometrically, and based on the equation of Moss et al. [41], the shift in the fermentation pattern observed in our study should have resulted in a reduction in methane of 10%.

Stevia extract is occasionally used in animal feeding mainly to improve the palatability of the feed and subsequently increase feed intake and body weight gain, particularly in chickens [42] and pigs [43]. Our interest in stevia extract stemmed from its 2,5 dihydroxymethyl-3,4-dihydroxypyrrolidine (DMDP) content, a potent glycosidase inhibitor that could prevent the deglycosylation of saponins to sapogenins in the rumen. The antimicrobial activity of stevia extract has been previously shown [44,45,46] and an antiprotozoal effect has been suggested [47]. Our results revealed that stevia extract also had an effect on protozoa decreasing protozoal activity by 84% when incubated at 2 g/L. In agreement with this observation, 24 h incubations with stevia extract resulted in a decrease (-80%) in protozoa concentration which was more pronounced than that caused by ivy saponins at 1 g/L. Stevia extract seemed to have a greater impact on *Entodinium*, which have been suggested to be responsible of the majority of bacterial protein turnover in the rumen [48]. Regarding fermentation parameters, incubations with stevia extract alone resulted in a decrease in ammonia (-44%) and butyric acid (-17%) concentrations, which could be related to the decrease in protozoa numbers, and an increase in propionic acid concentration (+35%). While we speculate that the observed effects are attributed to the high iminosugar content in stevia extract, it is important to note that other not yet identified components/phytochemicals may be contributing to the reported effect. Thus, it appears that stevia extract could be used on its own as a feed additive for ruminants to control protozoa and potentially modify rumen fermentation.

As we hypothesised, the combination of ivy with stevia extract caused a greater effect on protozoa and fermentation than that observed in incubations with ivy or stevia alone [49]. In the presence of ivy plus stevia, protozoa motility was reduced to a greater extent than when ivy or stevia were incubated alone (Fig 1). Ivy and stevia together were more effective in shifting the fermentation pattern towards higher propionate (+38.6%) and lower butyrate (-32.5%) production than ivy and stevia incubated separately. Ammonia concentration was also dramatically reduced (-64.5%) when both extracts were present.

The synthesized compound, HBS, showed the greatest antiprotozoal effect of the compounds tested causing the complete abolition of predatory activity, when added at 1 g/L. In *vitro* 24 h incubations with HBS resulted in a 76% decrease in protozoa concentration, similar to the decrease observed in the presence of stevia extract combined with ivy. Protozoa motility over time was, however, strongly affected by HBS; at 24 h no motility or ciliary activity was observed and the presence of vacuoles in most of the cells was detected, suggesting protozoal death. HBS enhanced ruminal fermentation, as reflected by an increase in VFA, promoting a decrease in butyrate molar proportion (-45%) which was compensated by an increase in propionate (+44%). HBS also resulted in a decrease in ammonia concentration (-42%) although to a lesser extent than that caused by ivy saponins, either alone or with stevia. If these effects can be replicated *in vivo*, the synthesised saponin could be potentially useful to boost milk production as increased propionate may enhance glucose synthesis by the animal [50].

In this experiment, we attempted to enhance the antiprotozoal effect of saponins in the rumen by preventing their deglycosylation to sapogenins either through chemical modification of the saponin (HBS) or through the addition of a glycosidase inhibitor in the form of stevia extract. However, examination of the bacterial population clearly showed an effect on bacteria in addition to protozoa. Multivariable analysis indicated that these changes in bacterial population structure correlated with changes in rumen fermentation parameters. Whilst it is possible that this was an indirect effect resulting from the change in protozoal numbers [1], further studies are required to examine the direct effect of the additives, and in particular stevia extract, on the bacterial population in the rumen.

## Conclusions

The antiprotozoal effect of ivy saponins either combined with an iminosugar-rich stevia extract or after chemical modification (HBS) was greater than that of ivy saponins. Combining the saponins with glycosidase inhibitors or changing the structure of the saponins to, ultimately, avoid deglycosylation resulted in different biological activities. Ivy saponins combined with stevia were more effective in reducing ammonia production. HBS had a greater effect on shifting fermentation towards propionate which was probably related to the changes in the structure and diversity of the bacterial community observed. Longer term studies are needed to confirm the observed effects as well as to elucidate the mode of action of ivy saponins combined with stevia and HBS *in vivo*.

## References

[1] Newbold CJ, de la Fuente G, Belanche A, Ramos-Morales E, McEwan NR. The role of ciliate protozoa in the rumen. Front Microbiol. 2015; 6: 1313 doi: 10.3389/fmicb.2015.01313 26635774 10.3389/fmicb.2015.01313 PMC4659874

[2] Patra KA, Saxena J. Dietary phytochemicals as rumen modifiers: a review of the effects on microbial population. Antonie van Leeuwenhoek 2009; 96: 363–375. doi: 10.1007/s10482-009-9364-1 19582589 10.1007/s10482-009-9364-1

[3] Francis G, Kerem Z, Makkar HPS, Becker K. The biological action of saponins in animal systems: a review. Br J Nutr. 2002; 88: 587–605. doi: 10.1079/BJN2002725 12493081 10.1079/BJN2002725

[4] Patra AK, Saxena J. The effect and mode of action of saponins on the microbial populations and fermentation in the rumen and ruminant production. Nutr Res Rev. 2009; 22: 204–219. doi: 10.1017/S0954422409990163 20003589 10.1017/S0954422409990163

[5] Newbold CJ, ElHassan SM, Wang J, Ortega ME, Wallace RJ. Influence of foliage from African multipurpose trees on activity of rumen protozoa and bacteria. Br J Nutr. 1997; 78: 237–249. 9301414 10.1079/bjn19970143

[6] Kohda H, Kasai R, Yamsaki K, Murakami K, Tanaka O. New sweet diterpene glucosides from *Stevia rebaudiana*. Phytochemistry 1976; 15: 981–983.

[7] Michalik A, Hollinshead J, Jones L, Fleet GWJ, Yu CY, Hu XG et al Steviamine, a new indolizidine alkaloid from Stevia rebaudiana. Phytochem Lett. 2010; 3: 136–138.

[8] Asano N. Naturally occurring iminosugars and related compounds: structure, distribution, and biological activity. Curr Top Med Chem. 2003; 3(5): 471–84. 12570862 10.2174/1568026033452438

[9] Nash RJ, Kato A, Yu CY, Fleet GWJ. Iminosugars as therapeutic agents: recent advances and promising trends. Future Med Chem. 2011; 3(12): 1513–1521. doi: 10.4155/fmc.11.117 21882944 10.4155/fmc.11.117

[10] Ramos-Morales E, de la Fuente G, Nash R, Preskett D, Newbold CJ. In vitro effect of the combination of Ivy fruit refined extract and a glucosidase inhibitor on rumen fermentation and protozoa activity. Rowett-INRA 2014, Gut Microbiology: from sequence to function 2014 p 116.

[11] Ramos-Morales E, de la Fuente G, Duval S, Wehrli C, Bouillon M, Lahmann M et al Antiprotozoal effect of saponins in the rumen can be enhanced by chemical modifications in their structure. Front. Microbiol. 2017; 8: 339 doi: 10.3389/fmicb.2017.00339 28382023 10.3389/fmicb.2017.00399 PMC5361656

[12] Pielartzik H, Irmisch-Pielartzik B, Eichler T. Carbonsäureester. In: Houben J, Weyl T and Müller E, editors. Methoden der organischen Chemie; 1985. pp. 656–715.

[13] Ramos-Morales E, Duval S, Wehrli C, Bouillon M, Preskett D, Braganca R, et al. New bis esters of Ivy sapogenins for ruminants; 2016. Patent PCT/EP2016062383.

[14] Nash RJ, Goldstein WS, Evans SV, Fellows LE. Gas chromatographic method for separation of nine polyhydroxy alkaloids. J Chromatogr. 1986; 366: 431–434.

[15] Wallace RJ, McPherson CA. Factors affecting the rate of breakdown of bacterial protein in rumen fluid. Br J Nutr. 1987; 58: 313–323. 3118940 10.1079/bjn19870098

[16] Williams AG, Coleman GS. The rumen protozoa. Springer-Verlag New York Inc New York; 1992.

[17] Morgavi DP, Boudra H, Jouany JP, Michalet-Doreau B. Effect and stability of gliotoxin, an Aspergillus fumigatus toxin, on in vitro rumen fermentation. Food Addit Contam. 2004; 21(9): 871–8. doi: 10.1080/02652030400002188 15666981 10.1080/02652030400002188

[18] Wallace RJ, McKain N, Shingfield KJ, Devillard E. Isomers of conjugated linoleic acids are synthesized via different mechanisms in ruminal digesta and bacteria. J Lipid Res. 2007; 48(10): 2247–2254. doi: 10.1194/jlr.M700271-JLR200 17644775 10.1194/jlr.M700271-JLR200

[19] Menke KH, Steingass H. Estimation of the energetic feed value obtained from chemical analysis and gas production using rumen fluid. Anim Res Dev. 1988; 28: 7–55.

[20] Newbold CJ. Assessing antiprotozoal agents. In: Vercoe PE, Makkar HPS and Schilink C, editors. In vitro Screening of plant resources for extra-nutritional attributes in ruminants; 2010. pp. 47–53.

[21] Stewart CS, Duncan SH. The effect of avoparcin on cellulolytic bacteria of the ovine rumen. J Gen Microbiol. 1985; 131: 427–435.

[22] Weatherburn MW. Phenol-hypochlorite reaction for determination of ammonium. Anal Chem. 1967; 39: 971–974.

[23] Dehority BA. Laboratory manual for classification and morphology of ruminal ciliate protozoa. United States: CRC Press, Boca Raton, FL; 1993.

[24] de la Fuente G, Skirnisson K, Dehority BA. Rumen ciliate fauna of Icelandic cattle, sheep, goats and reindeer. Zootaxa 2006; 1377: 47–60.

[25] Pitchern DG, Saunders A, Owen RJ. Rapid extraction of bacterial genomic DNA with guanidium thiocyanate. Lett Appl Microbiol. 1989; 8: 151–156.

[26] Boom R, Sol CJA, Salimans MMM, Jansen CL, Wertheim-Van Dillen PME, Van DerNoordaa J. Rapid and Simple Method for Purification of Nucleic Acids. J Clin Microbiol. 1990; 28(3): 495–503. 1691208 10.1128/jcm.28.3.495-503.1990 PMC269651

[27] Hongoh Y, Yuzawa H, Ohkuma M, Kudo T. Evaluation of primers and PCR conditions for the analysis of 16S rRNA genes from a natural environment. FEMS Microbiol Lett. 2003; 221: 299–304. 12725942 10.1016/S0378-1097(03)00218-0

[28] Skrivanová E, Worgan HJ, Pinloche E, Marounek M, Newbold CJ, McEwan NR. Changes in the bacterial population of the caecum and stomach of the rabbit in response to addition of dietary caprylic acid. Vet Microbiol. 2010; 144: 334–339. doi: 10.1016/j.vetmic.2010.01.013 20181443 10.1016/j.vetmic.2010.01.013

[29] Hill TC, Walsh KA, Harris JA, Moffett BF. Using ecological diversity measures with bacterial communities. FEMS Microbiol Ecol. 2003; 43(1): 1–11. doi: 10.1111/j.1574-6941.2003.tb01040.x 19719691 10.1111/j.1574-6941.2003.tb01040.x

[30] Benjamini Y, Hochberg Y. Controlling the false discovery rate—a practical and powerful approach to multiple testing. J Roy Stat Soc B Met. 1995; 57: 289–300.

[31] Hu WL, Liu JX, Ye JA, Wu YM, Guo YQ. Effect of tea saponin on rumen fermentation *in vitro*. Anim Feed Sci Technol. 2005; 120: 333–339.

[32] Guo YQ, Liu JX, Lu Y, Zhu WY, Denman SE, McSweeney CS. Effect of tea saponin on methanogenesis, microbial community structure and expression of mcrA gene, in cultures of rumen microorganisms. Lett Appl Microbiol. 2008; 47: 421–426. doi: 10.1111/j.1472-765X.2008.02459.x 19146532 10.1111/j.1472-765X.2008.02459.x

[33] Wina E, Muetzel S and Becker K. The impact of saponins or saponin-containing plant materials on ruminant production- A review. J Agric Food Chem. 2005; 53: 8093–8105. doi: 10.1021/jf048053d 16218650 10.1021/jf048053d

[34] Uddin G, Rauf A, Qaisar M, Rehman TU, Latif A, Ali M. Preliminary Phytochemical Screening and Antimicrobial Activity of *Hedera Helix* L. Middle East J Sci Res. 2011; 8(1): 198–202.

[35] Parvu M, Vlase L, Parvu AE, Rosca-Casian O, Gheldiu AM, Parvu O. Phenolic compounds and antifungal activity of Hedera helix L. (Ivy) flowers and fruits. Not Bot Horti Agrobo. 2015; 43(1): 53–58.

[36] Lutsenko Y, Bylka W, Matlawska I, Darmohray R. Hedera helix as a medicinal plant. Herba Pol. 2010; 56(1): 83–96.

[37] Belanche A, Pinloche E, Preskett D, Newbold CJ. Effects and mode of action of chitosan and ivy fruit saponins on the microbiome, fermentation and methanogenesis in the rumen simulation technique. FEMS Microbiol Ecol, 2016; 92 doi: 10.1093/femsec/fiv160 26676056 10.1093/femsec/fiv160 PMC5831848

[38] Belanche A, Ramos-Morales E, Newbold CJ. In vitro screening of natural feed additives from crustaceans, diatoms, seaweeds and plant extracts to manipulate rumen fermentation. J Sci Food Agric. 2016; 96: 3069–3078. doi: 10.1002/jsfa.7481 26441121 10.1002/jsfa.7481

[39] Tedeschi LO, Fox DG, Russell JB. Accounting for ruminal deficiencies of nitrogen and branched-chain amino acids in the structure of the Cornell net carbohydrate and protein system Proceedings of Cornell Nutrition Conference for Feed Manufacturers; New York: Cornell University; 2000.

[40] Jayanegara A, Wina E, Takahashi J. Meta-analysis on methane mitigation properties of saponin-rich sources in the rumen: Influence of addition levels and plant sources. Asian Australas J Anim Sci. 2014; 27: 1426–1435. doi: 10.5713/ajas.2014.14086 25178294 10.5713/ajas.2014.14086 PMC4150175

[41] Moss AR, Jouany JP, Newbold CJ. Methane production by ruminants: its contribution to global warming. Ann Zootech. 2000; 49 (3): 231–253.

[42] Atteh J, Onagbesan O, Tona K, Decuypere E, Geuns J, Buyse J. Evaluation of supplementary Stevia (Stevia rebaudiana Bertoni) leaves and stevioside in broiler diets: Effects on feed intake, nutrient metabolism, blood parameters and growth performance. J Anim Physiol and Anim Nut. 2008; 92: 640–649. 10.1111/j.1439-0396.2007.00760.x 19012609

[43] Wang LS, Shi Z, Shi BM, Shan AS. Effects of dietary stevioside/rebaudioside A on the growth performance and diarrhoea incidence of weaned piglets. Anim Feed Sci Technol. 2014; 187: 104–109.

[44] Tomita T, Sato N, Arai T, Shiraishi H, Sato M, Takeuchi M et al Bactericidal activity of a fermented hot-water extract from Stevia rebaudiana Bertoni towards enterohemorrhagic Escherichia coli O157:H7 and other food-borne pathogenic bacteria. Microbiol. Immunol. 1997; 41: 1005–1009. 9492187 10.1111/j.1348-0421.1997.tb01961.x

[45] Debnath M. Clonal propagation and antimicrobial activity of an endemic medicinal plant Stevia rebaudiana. J Med Plants Res. 2008; 2: 45–51.

[46] Ghosh S, Subudhi E, Nayak S. Antimicrobial assay of Stevia rebaudiana Bertoni leaf extracts against 10 pathogens. Int J Integr Biol. 2008; 2: 27–31.

[47] Torres M, Ortega O, Báez M, González A, Lara M, Sardi S. Effects of *Stevia rebaudiana* (KA'A HE'E) on the rumen metabolism indicators in sheep fed gunned fattening. Compend. Cienc. Vet. 2015; 5: 32–37.

[48] Belanche A, de la Fuente G, Moorby JM, Newbold CJ. Bacterial protein degradation by different rumen protozoal groups. J Anim Sci. 2012; 90: 4495–4504. doi: 10.2527/jas.2012-5118 22829613 10.2527/jas.2012-5118

[49] Ramos-Morales E, Nash R, Braganca R, Newbold CJ. Methods and Compositions for improving feed outcomes; 2016. UK patent application number 1620438.0.

[50] Seymour WM, Campbell DR, Johnson ZB. Relationships between rumen volatile FA concentrations and milk production in dairy cows: A literature study. Anim Feed Sci Tech. 2005; 119: 155–169.

